# Controlled Growth of CdS Nanostep Structured Arrays to Improve Photoelectrochemical Performance

**DOI:** 10.3389/fchem.2020.577582

**Published:** 2020-12-10

**Authors:** Jiangang Jiang, He Wang, Hongchang An, Guangyuan Du

**Affiliations:** College of Science, Northwest Agriculture and Forestry University, Shaanxi, China

**Keywords:** nanostep, morphology control, photoelectrode, film, water splitting

## Abstract

CdS nanostep-structured arrays were grown on F-doped tin oxide-coated glasses using a two-step hydrothermal method. The CdS arrays consisted of a straight rod acting as backbone and a nanostep-structured morphology on the surface. The morphology of the samples can be tuned by varying the reaction parameters. The phase purity, morphology, and structure of the CdS nanostep-structured arrays were characterized by X-ray diffraction and field emission scanning electron microscopy. The light and photoelectrochemical properties of the samples were estimated by a UV-Vis absorption spectrum and photoelectrochemical cells. The experimental results confirmed that the special nanostep structure is crucial for the remarkable enhancement of the photoelectrochemical performance. Compared with CdS rod arrays, the CdS nanostep-structured arrays showed increased absorption ability and dramatically improved photocurrent and energy conversion efficiency. This work may provide a new approach for improving the properties of photoelectrodes in the future.

## 1. Introduction

There is a high demand for a sustainable energy source as fossil fuel consumption continues to cause environmental harm. Among the various substitutes for fossil fuel, solar energy is considered to be an ideal candidate because it is inexhaustible, clean, and widely distributed globally (Hisatomi et al., 2014; Ning et al., 2017; Zhang et al., 2020). However, the widespread application of solar energy continues to be a challenge due to its discontinuity (Low et al., 2017; Zhang et al., 2018). To address this issue, great efforts have been made to achieve solar energy conversion and storage. For instance, photoelectrochemical (PEC) water splitting using solar energy aims at converting solar energy into hydrogen and oxygen to create a renewable energy system (Courtin et al., 2014; Ahmed and Dincer, 2019). This technology builds a bridge between solar energy and hydrogen, which is considered green energy, prompting great interest among researchers (Fang et al., 2017; Qi et al., 2018; Hirscher et al., 2020). The key factor in PEC water splitting is the development of highly efficient photoelectrodes based on semiconductors (Jiang C. et al., 2017; Chen et al., 2018).

Many kinds of photoelectrodes, such as Fe_2_O_3_, BiVO_4_, WO_3_, and CdS (Ji et al., 2016; Sun et al., 2018; Fu et al., 2019; Wang et al., 2019), have been developed since the Fujisha and Hoda reported that TiO_2_ could be used as a photoelectrode for solar energy conversion (Fujishima and Honda, 1972). There is particular interest in CdS due to its suitable band gap and high absorption coefficient, which results in relatively efficient PEC and photocatalytic performance (Cheng et al., 2018). CdS is a well-known narrow band gap semiconductor that is widely used in lasers, light-emitting diodes, and solar cells (Zhang et al., 2016; Zapf et al., 2017; Bosio et al., 2018). Many researchers have focused on the application of CdS in the field of PEC/photocatalytic water splitting for hydrogen production. For example, Li's group reported an exceptionally high quantum efficiency (93%) of photocatalytic hydrogen production on Pt–PdS/CdS (Yang et al., 2012). Liu et al. (2013) obtained 62% quantum efficiency of solar hydrogen evolution over Cd_0.5_Zn_0.5_S without noble metal loading. After further experimentation, they achieved an internal quantum efficiency approaching 100% at 425 nm based on Cd_0.5_Zn_0.5_S with NiS_*x*_ as co-catalyst (Liu et al., 2016). All these studies demonstrated that CdS is a promising semiconductor for high solar energy conversion. Nevertheless, pure CdS still possesses inherent drawbacks, such as rapid charge recombination and poor stability in solution, which limits its practical application (Yan et al., 2009; Xie et al., 2014).

Various strategies have been explored to address the disadvantages of CdS to promote charge carrier separation and to enhance the efficiency of solar energy conversion (Zhang and Lou, 2019; Zheng et al., 2019). Heterojunction design is a common method that has been widely used to improve the separation of photogenerated electron-hole pairs (Li et al., 2018; Wu et al., 2019). Doping CdS with metal or non-metal elements is another measure used to tune the energy band structure and enhance the properties of CdS (Lee et al., 2016). In addition to the above methods, morphology control can be used to improve the PEC performance of CdS (Vaquero et al., 2017). For example, Jing synthesized CdS particles with screw-thread-like nanostep structures and demonstrated that this structure is crucial for enhancing photocatalytic hydrogen production (Jing and Guo, 2006). Chen prepared CdS nanorod arrays without a template and demonstrated their potential applications in optoelectronics (Chen et al., 2011). Liu et al. fabricated porous flower-like, belt-like, and net-like CdS photocatalysts using a mixed-solvothermal strategy. The flower-like CdS exhibited the highest photocatalytic activity for H_2_ evolution under visible light without any co-catalyst (Liu et al., 2018). Each of the above studies demonstrated a promising approach to improve solar energy conversion based on the morphology control of CdS.

Herein, we report a two-step hydrothermal method to synthesize nanostep arrays as photoelectrodes for improved PEC performance. The CdS arrays consist of a straight rod as the backbone and a nanostep-structured morphology on the surface. The morphology and PEC performance of the samples can be tuned by varying the reaction parameters. Experimental results show that, compared to the sample without the nanostep structure, the CdS nanostep arrays exhibited better PEC performance. This work may provide a new approach for improving the properties of photoelectrodes in the future.

## 2. Experimental Section

### 2.1. Raw Materials

All chemical agents were of analytical grade and were used without further treatment. F-doped tin oxide (FTO)-coated glass as substrates (15Ω/square) were purchased from Nippon Sheet Glass Co., Ltd. Acetone (C_3_H_6_O), absolute ethanol (C_2_H_6_O), hydrochloric acid (HCl), cadmium nitrate (Cd(NO_3_)_2_·4H_2_O), thiourea (CS(NH_2_)_2_), and glutathione (GSH) were purchased from Sinopharm Chemical Reagent Limited Corporation. Deionized water was used in all experiments. For synthesis of CdS films on FTO substrates, the FTO substrates were first ultrasonically cleaned in deionized water, acetone, and absolute ethanol alternatively 15 min per step.

### 2.2. Synthesis of CdS Nanorod Arrays

CdS nanorod arrays were deposited on the cleaned FTO substrate using a hydrothermal method. In a typical experiment, cadmium nitrate (1 mmol), thiourea (3 mmol), and GSH (0.6 mmol) were dissolved in 80 mL deionized water. This solution was poured into Teflon lined stainless steel autoclave containing an FTO glass substrate placed at an angle and partially immersed into the solution. Then, the autoclave was transferred to electricity heat drum wind drying oven and maintained 200°C for 2, 4, and 6 h to determine the optimal reaction time. Finally, the sample was removed from the autoclave and rinsed with deionized water after the autoclave cooled naturally. The obtained samples were denoted as CdS-2h, CdS-4h, and CdS-6h, respectively.

### 2.3. Synthesis of CdS Nanostep-Structured Arrays

As the sample prepared for 4 h showed the best PEC performance, this sample was used thereafter. CdS nanostep arrays were synthesized by a second hydrothermal approach that was completed by keeping the sample CdS-4h in the autoclave at 200°C for 1, 2, 3, and 4 h with same precursor solution. Finally, the obtained samples were rinsed with deionized water. The samples were denoted as CdS-T-1h, CdS-T-2h, CdS-T-3h, and CdS-T-4h, respectively. To further improve the PEC performance of CdS nanostep arrays, CdS-4h was first treated in 3.7 wt% HCl solution for 30 s before second hydrothermal process. Subsequently, the second hydrothermal process was completed by keeping treated CdS-4h in the autoclave at 200°C for 3 h with same precursor solution. The obtained sample was denoted as CdS-HT-3h.

### 2.4. Characterization

The sample morphology was observed using a JEOL JSM-7800 scanning electron microscopy (SEM). X-ray diffraction (XRD) patterns were obtained on a PANalyticalX'pert MPD Pro X-ray diffractometer using Ni-filtrated Cu K_α_ irradiation (wavelength 1.5406°*A*). The optical spectra of the samples were determined with a Hitachi U-4100 UV-vis-near-IR spectrophotometer using BaSO_4_ as the reference. Linear sweep voltammetry (LSV) under chopped light illumination was conducted using an electrochemical workstation (CHI 760D) in a three-electrode system.

### 2.5. PEC Measurements

PEC measurements were carried out in a convenient three electrodes cell. Work electrodes were made up of the sample films. The work electrodes were mounted onto a special designed electrode holder and surface areas exposed to electrolyte were fixed at 0.785 cm^2^. A saturated calomel electrode (SCE) was used as a reference electrode, and a large area platinum plate was used as a counter electrode. An aqueous solution of 0.5 M Na_2_SO_3_ was prepared as the electrolyte. An electrochemical workstation (CHI 760D) from CH Instruments was used for photocurrent measurements under 100 mW/cm^2^ chopped light illumination. The scanning rate was 10 mV/s, and the scanning direction was from low to high potential. The absolute intensity of the incident light was recorded with an avaspec-2048 fiber optical spectrometer from Avnantes.

## 3. Results and Discussion

**Figure 1:**
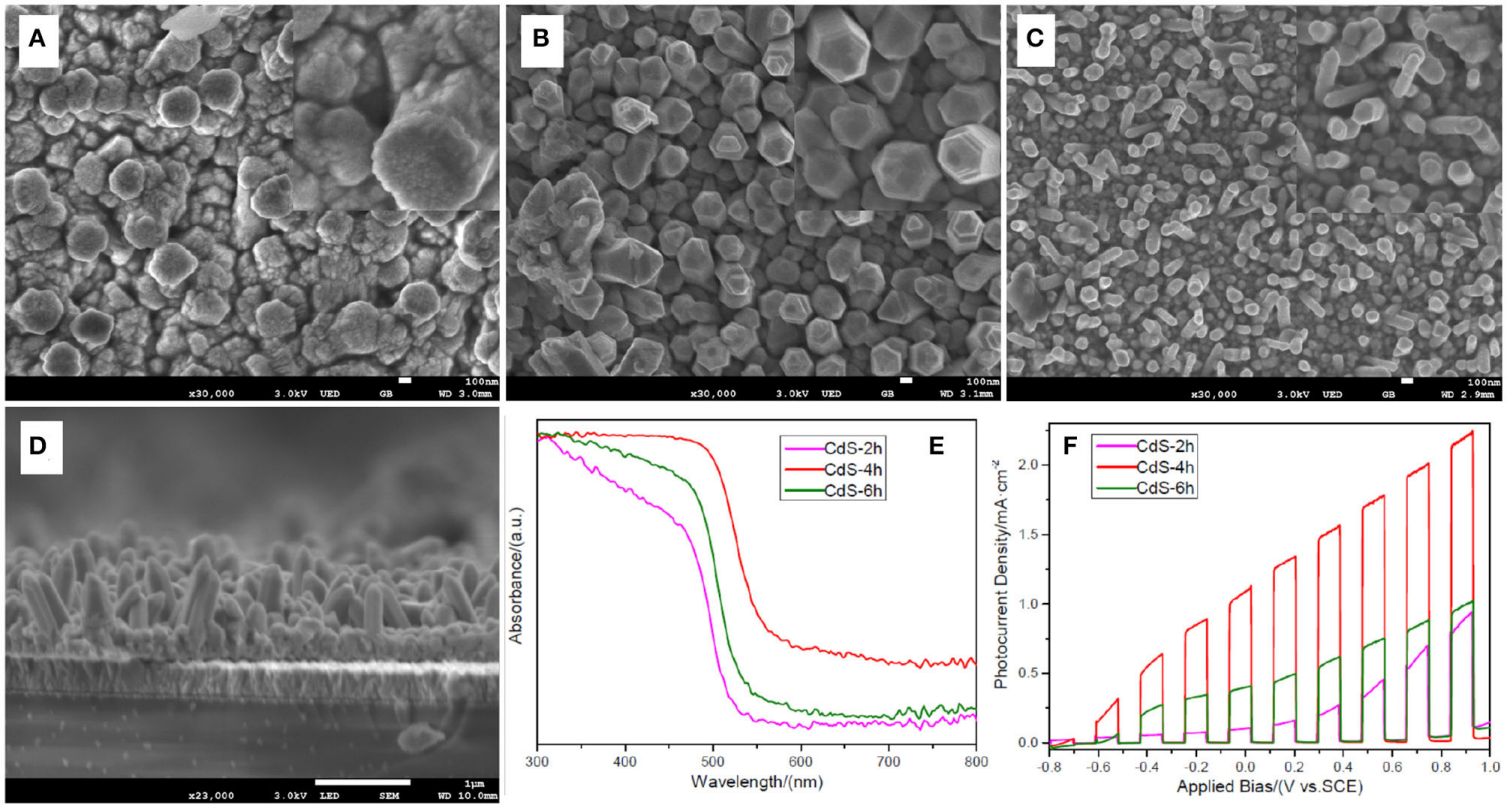
Scanning electron microscopy (SEM) images of **(A)** sample CdS-2h; **(B)** sample CdS-4h; **(C)** sample CdS-6h; **(D)** crossing-section view of sample CdS-4h; insert pictures are high-magnification SEM image of CdS-2h, CdS-4h, and CdS-6h; **(E)** Uv-Vis absorbance of CdS-2h, CdS-4h, and CdS-6h; **(F)** linear sweep voltammetry (LSV) curves of CdS-2h, CdS-4h, and CdS-6h under chopped light illumination.

Figure 1 shows the SEM images of samples prepared through a one-step hydrothermal approach maintained at 200°C for 2, 4, and 6 h. As shown in Figure 1A, a few irregular rods made up of massive nanoparticles were formed. When the hydrothermal time was increased to 4 h, the morphology of the sample changed considerably. The apparent nanorod arrays without nanoparticles are shown in Figure 1B. Through careful observation of the insert picture in Figure 1B, a frustum-like structure can be found at the top of the nanorod arrays, which may relate to the acidity of the precursor. This will be discussed in the following sections. The diameter of the nanorods decreased gradually as the reaction time increased from ~200 nm for 4 h to ~100 nm for 6 h. The thickness (~1 μm) of film fabricated for 4 h is shown in the SEM image of crossing section (Figure 1D). The image also shows that nanorod arrays uniformly disperse on the FTO surface. The (002) peak is higher in intensity compared with other peaks in the XRD patterns (Supplementary Figure 1). This further confirms the synthesis of CdS nanorod arrays through a one-step hydrothermal method at 200°C for 4 and 6 h.

Generally, the light absorption and PEC performance of the samples are the primary concerns for energy conversion. Figure 1E shows the absorbance of the three samples that possess the specific absorption property of CdS (Ai et al., 2018). For the sample prepared for 2 h, the absorption edge is approximately 520 nm, corresponding to 2.38 eV in the band gap as calculated by the equation Eg = 1,240/λ (Humayun et al., 2018). When the hydrothermal time was increased, the absorption edge of the sample shifted to 560 nm (4 h) and then moved back to 540 nm (6 h), corresponding to 2.21 and 2.30 eV in the band gap. The changes in the band gap of the samples may be influenced by the crystal size, i.e., quantum confinement effect (Chen et al., 2019). In Figure 1A, it is clear that the sample prepared for 2 h is composed of many particles that are the smallest in size of the three samples. Therefore, it exhibits the largest band gap. The sample prepared for 4 h has the smallest band gap because of its large crystal size, which is beneficial for PEC performance. This is because a semiconductor with a smaller band gap can absorb light energy in a wider range of the solar spectrum (Samsudin and Abd Hamid, 2017). Upon further observation of the absorption curve, it is notable that the absorption of sample CdS-4h is dramatically stronger than that of other two samples, particularly in wavelengths between 500 and 800 nm. The enhancement of the absorption capacity is ascribed to the multi-scattering and light-trapping effect created by the nanorod arrays. (Cho et al., 2011). Based on the above analysis, it is expected that the sample CdS-4h would have better PEC performance, and this was confirmed by the PEC results shown in Figure 1F. The photocurrent density of the sample CdS-4h is higher than that of the other two samples in the entire applied bias region, reaching a value of 1.08 mA/cm^2^ at 0 V, 10 times that of the sample prepared for 2 h. The significant improvement in PEC performance may be due to better light absorption capability and facile transportation of chargers arising from straight CdS nanorods (Wang W. et al., 2018).

**Figure 2:**
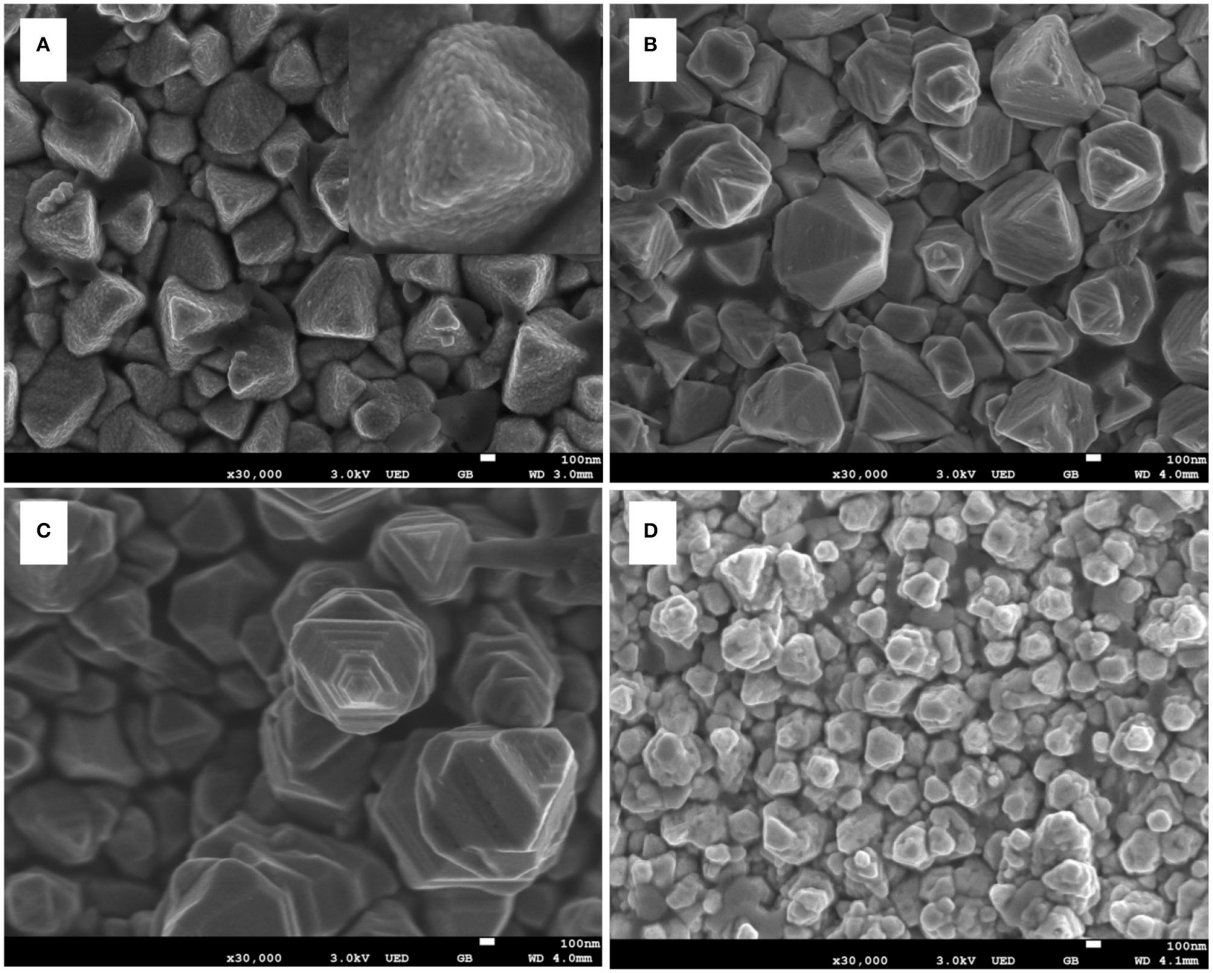
Scanning electron microscopy (SEM) images of **(A)** sample CdS-T-1h; **(B)** sample CdS-T-2h; **(C)** sample CdS-T-3h; **(D)** sample CdS-T-4h; insert picture is high-magnification SEM image of CdS-T-1h.

As the sample prepared for 4 h showed the best PEC performance (Figure 1F), this sample was used as the base to synthesize CdS nanostep arrays. Figure 2 shows the SEM images of samples that were prepared by second step hydrothermal approach at 200°C for 1, 2, 3, and 4 h. As shown in Figure 2A and in the inset picture, bulky pyramid-like rods are composed of tiny particles with ~10 nm in diameter, and no nanostep CdS arrays were found. When the second hydrothermal time was increased to 2 h, polycrystalline CdS rods transformed into pyramidal and frustum-like rods (Figure 2B). This transformation was also found by Li's group when the reaction time was increased above 1 h (Yang et al., 2013). Upon further increasing the reaction time to 3 h, an obvious nanostep structure was formed on the surface of the CdS rods. The nanostep structure may be beneficial for enhancing the light absorption due to the multi-scattering and reflection effect of light (Bera et al., 2018). However, the nanostep structure disappeared as the time was extended to 4 h. In this case, the CdS rod was transformed into a thinner and irregular rod (Figure 2D), only ~100 nm in diameter, in contrast with the nanostep structural rod with a ~600 nm in diameter. The evolution of the rod diameter with time is also observed in Figures 1A–C. This change in rod diameter and morphology may be caused by the synergistic effect of GSH used as a capping agent and the acidity of the precursor solution. Li's and Chen's group reported that thiol and dicarboxylic groups in GSH played a vital role in forming CdS crystal particles and nanorod arrays. The thiol and dicarboxylic groups may selectively absorb on the low-index faces of CdS, leading to slow growth along that side and then forming CdS nanorods (Chen et al., 2008; Yang et al., 2013). The CdS rod may be etched during the hydrothermal approach due to the acidity of the hydrothermal solution containing GSH as an acidic polypeptide (Tummanapelli and Vasudevan, 2015). Under these conditions, the high-energy face of the CdS rod could be etched first to shape the nanostep structure on the rod surface. The etching process will be continued with hydrothermal time, eventually resulting in a reduction in the diameter of the rods. Thus, there is a specific amount of time needed to obtain a CdS rod with a nanostep structure.

**Figure 3:**
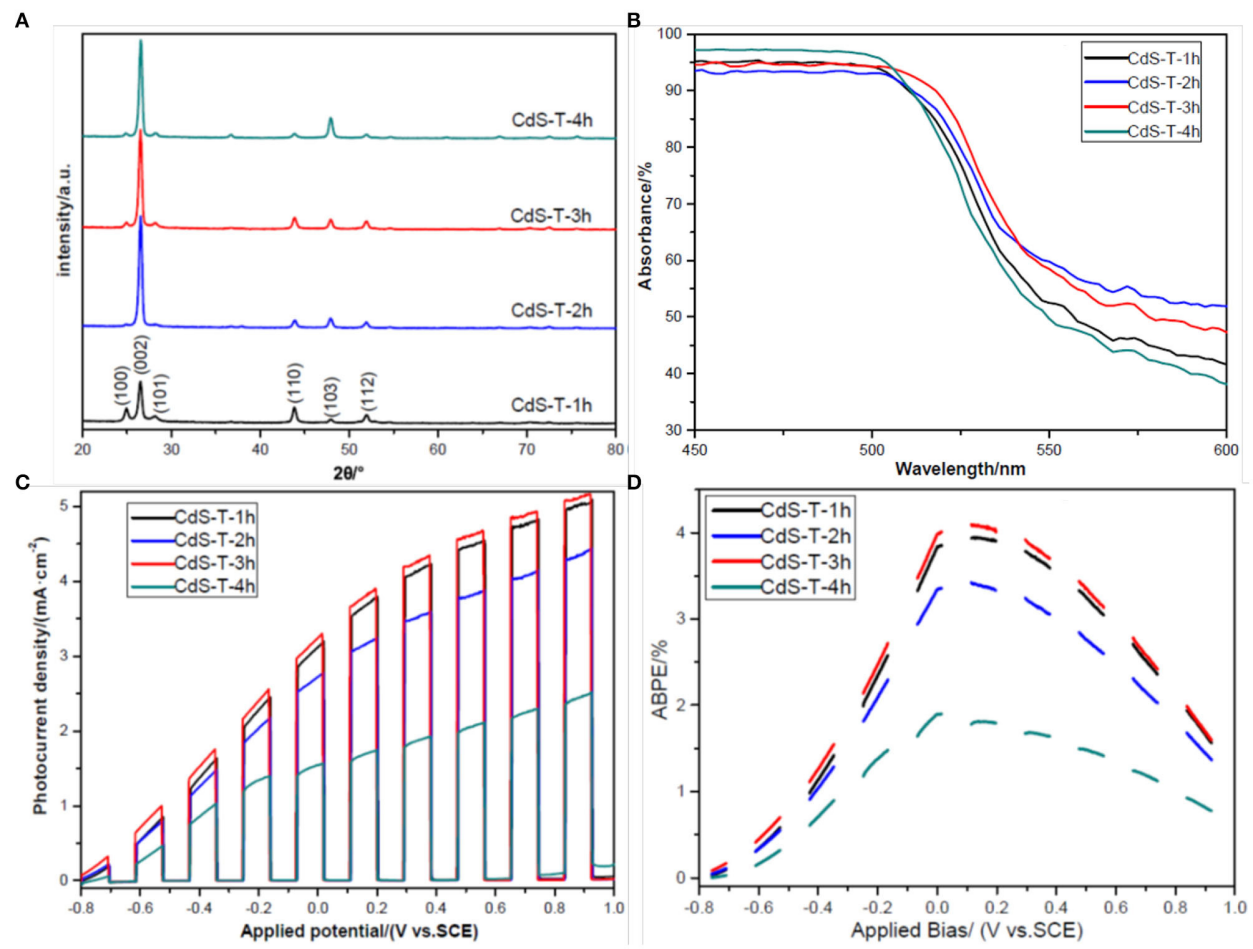
**(A)** X-ray diffraction (XRD) patterns of samples CdS-T-1h, CdS-T-2h, CdS-T-3h, and CdS-T-4h; **(B)** Uv-Vis absorbance of samples CdS-T-1h, CdS-T-2h, CdS-T-3h, and CdS-T-4h; **(C)** linear sweep voltammetry (LSV) curves of samples CdS-T-1h, CdS-T-2h, CdS-T-3h, and CdS-T-4h under chopped light illumination; **(D)** applied bias photon-to-current efficiency (ABPE) curves of samples CdS-T-1h, CdS-T-2h, CdS-T-3h, and CdS-T-4h under chopped light illumination.

The XRD patterns of the as-prepared samples are shown in Figure 3A. It is evident that all diffraction peaks correspond to the hexagonal wurtzite CdS phase (JCPDS No. 77-2306) (Shengyuan et al., 2012). There are no other peaks to be found, demonstrating the purity of all CdS samples synthesized by a two-step hydrothermal process. Furthermore, the differences between the samples can be confirmed by comparing the peak intensity of the (002) facet. The peak intensity of the (002) facet has an overwhelming advantage over other peaks for the CdS-T-2h, CdS-T-3h, and CdS-T-4h, but not for CdS-T-1h, indicating preferential growth along the [002] direction for the CdS-T-2h, CdS-T-3h, and CdS-T-4h (Jiang J. et al., 2017). Considering the SEM images in Figure 2, it can be concluded that CdS particles are formed first after a short reaction time, and the polycrystalline CdS will gradually transform into CdS rods when the reaction time increases.

Figure 3B presents the light diffuse reflectance spectra of samples with different morphologies. To better distinguish these curves, we only show the spectrum between 450 and 600 nm in wavelength (the spectrum between 300 and 800 nm in wavelength is also presented in the Supporting Information, Supplementary Figure 2). As with other's reports (Bu et al., 2013; Wei et al., 2017), all curves show good visible light absorption of CdS, and the absorption edge is approximately 550 nm, corresponding to 2.25 eV in the band gap. Nevertheless, there is a slight difference between all spectrums. It was found that sample CdS-T-3h exhibits better absorption properties than the other samples, which may be ascribed to the rod arrays with a nanostep structure, as discussed in the previous section. Under these conditions, it is expected that sample CdS-T-3h may have better PEC performance than the other samples. The PEC performance of the samples is displayed in Figure 3C. The photocurrent density of all samples increased steadily with increasing applied bias, and gradually reached saturation, indicating the efficient separation of photogenerated chargers in the film (Wang F. et al., 2018). As expected, the photocurrent density of sample CdS-T-3h is highest in all samples at whole applied bias range and is approximately two times that of sample CdS-T-4h at 0 V (vs. SCE). Furthermore, the applied bias photon-to-current efficiency (ABPE), defined in Equation (1), is developed to characterize the energy conversion efficiency under an applied bias (Chen et al., 2010):

(1) $$\begin{array}{l} ABPE\left(\%\right)=\frac{j_{ph}\left(mA/cm^{2}\right)\times \left[1.23-|V_{b}|\right]\left(V\right)}{P_{total}\left(mW/cm^{2}\right)}\times 100 \end{array}$$

where j_*ph*_ is the photocurrent density obtained under an applied bias V_*b*_. P_*total*_ is incident illumination power density. As the ABPE curves shown in Figure 3D demonstrate, the ABPE value of all samples increased with increasing applied bias, indicating that the separation of chargers is generated by radiation. The ABPE value reaches a maximum at 0.1 V (vs. SCE), 4.09% for CdS-T-3h, and 1.77% for CdS-T-4h, confirming again that CdS-T-3h has the best PEC performance among the samples. The improvement in the PEC performance of the sample CdS-T-3h may be attributed to its unique rod arrays with a nanostep structure. The rod arrays provide a direct path for the transportation of chargers (Tak et al., 2009). The nanostep structure not only enlarges the contact area between the film and the electrolyte (Iwase et al., 2004), but also enhances the separation of chargers, which promotes surface reaction and improves the PEC performance of the samples (Shi et al., 2014; Cai et al., 2017).

**Figure 4:**
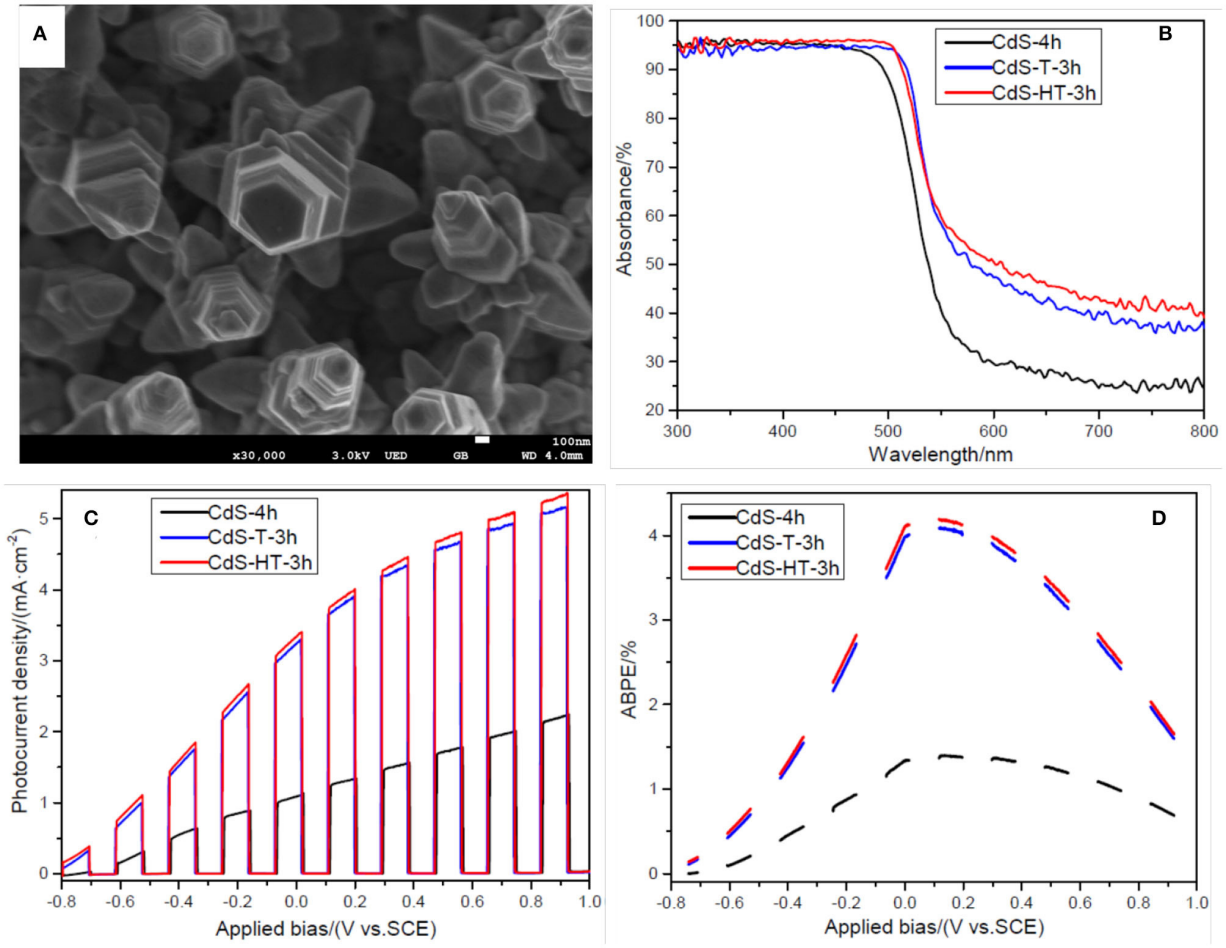
**(A)** Scanning electron microscopy (SEM) image of sample CdS-HT-3h; **(B)** Uv-Vis absorbance of samples CdS-4h, CdS-T-3h, and CdS-HT-3h; **(C)** LSV curves of samples CdS-4h, CdS-T-3h, and CdS-HT-3h under chopped light illumination; **(D)** applied bias photon-to-current efficiency (ABPE) curves of samples CdS-4h, CdS-T-3h, and CdS-HT-3h under chopped light illumination.

In order to further improve the PEC performance of the sample, CdS-4h was immersed in 3.7 wt% HCl solution for 30 s before second hydrothermal process in order to remove the organic group that may absorb to the film surface during the first hydrothermal process. Then, CdS-4h treated with hydrochloric acid was placed in an autoclave containing the same precursor at 200°C for 3 h. The obtained sample is denoted as CdS-HT-3h, and the SEM image of the CdS-HT-3T is shown in Figure 4A. A noticeable nanostep structural morphology is observed in the SEM image, similar to sample CdS-T-3h. In addition, large branches were distributed around the CdS backbone, forming a three-dimensional CdS structure, which could further enhance the light absorption of the sample (Dinh et al., 2014). Figure 4B provides evidence that CdS nanostep arrays with branches show slightly better light absorption in the spectral range of 300–800 nm, except for 510–540 nm, compared to CdS-T-3h. Exceptions at 510–540 nm may originate from the difference in crystal size between CdS-HT-3h and CdS-T-3h. With hydrochloric acid treatment, the sample CdS-HT-3h has a relatively smaller rod diameter than that of CdS-T-3h (compare Figure 2C with Figure 4A), which leads to a blue shift of the absorption edge discussed in the previous section. Subsequently, the LSV performance under chopped light was compared for the following structures: CdS rod arrays, CdS rod arrays with nanostep, and CdS rod arrays with nanostep and branches, as shown in Figure 4C. It is evident that CdS-HT-3h and CdS-T-3h both show significantly improved PEC performance when compared to the sample CdS-4h. The photocurrent density reaches 3.34 mA/cm^2^ at 0 V (vs. SCE) for CdS-HT-3h and 3.24 mA/cm^2^ for CdS-HT-3h, 3.3 and 3.2 times that of CdS-4h without nanostep structure. We also examined the ABPE curves of the three samples to further study energy conversion, and we found that they again confirm the advantage of the sample with the nanostep structure.

It is well-known that the PEC performance of a sample is highly dependent on three factors: the efficiency of charge generation, the efficiency of charger collection (transfer) at the electrode/electrolyte interface, and the efficiency of charge transport within the film (Ren et al., 2016). The efficiency of charge generation is closely related to the light absorption ability. The efficiency of charger collection and the efficiency of charge transport are related to the structure and electrical properties of the semiconductor. In the present experiment, the CdS rod arrays with a nanostep structure enhanced the light absorption arising from multiple reflection effects (Sun et al., 2014). Compared to the sample CdS-4h without the nanostep structure, both CdS-HT-3h and CdS-T-3h with a nanostep structure have large surface-to-volume ratios that provide sufficient reaction sites. Additionally, the photogenerated electrons and holes can readily migrate to the edge and groove sites of the nanostep structure on the surface of the CdS arrays due to the different charge densities between the edge and groove sites (Ding et al., 2018). These two factors increase the efficiency of charge separation and collection at the electrode/electrolyte interface. In terms of the efficiency of charge transport, the CdS rod arrays supply a direct pathway for photoinduced carrier transportation (Zhao et al., 2015; Qiu et al., 2019; Xu et al., 2020). All the above factors improve the PEC performance and enhance the light energy conversion of the sample. It is worth noting that sample CdS-HT-3h with branches on the surface only slightly improved its PEC performance compared to sample CdS-T-3h, which may not be consistent with our previous expectations. However, this is not the aim of this article. The goal of this work is to provide a new approach for synthesizing rod arrays with a nanostep structure on the surface. Based on this unique structure, the PEC performance of the photoelectrode can be continuously improved.

## 4. Conclusion

CdS rod arrays with a nanostep structural surface were grown on FTO-coated glasses through a two-step hydrothermal method. The morphology of the samples can be tuned by varying the reaction parameters, such as hydrothermal time and surface treatment. Compared to CdS rod arrays without a nanostep structure, the CdS nanostep structural arrays showed enhanced absorption ability and dramatically improved photocurrent and energy conversion efficiency, both of which contributed to the multiple reflection effect of light in the arrays and the enhanced charge transportation and collection.

## Data Availability Statement

The raw data supporting the conclusions of this article will be made available by the authors, without undue reservation.

## Author Contributions

JJ designed the experiments. HW carried out the experiments. HA analyzed the experimental results. GD gave helpful discussions to the conclusions. JJ wrote the manuscript. All authors contributed to the article and approved the submitted version.

## Conflict of Interest

The authors declare that the research was conducted in the absence of any commercial or financial relationships that could be construed as a potential conflict of interest.
