# Supplementary material for: Controlled Growth of CdS Nanostep Structured Arrays to Improve Photoelectrochemical Performance
Source: Front Chem. 2020 Dec 10;8:577582. doi: 10.3389/fchem.2020.577582 (PMC7758423; doi:10.3389/fchem.2020.577582)
Supplement: Supplementary file 1 [file Data_Sheet_1.PDF]

Supplementary Material

1 SUPPLEMENTARY DATA

This is the supporting information of manuscript titled "Controlled Growth of CdS Nanostep Structured Arrays to Improve Photoelectrochemical Performance"

2 SUPPLEMENTARY FIGURE

2.1 Figures

XRD pattern in Fig. S1 displays the wurtzite structure for the CdS nanorod arrays. Compared to CdS-2h and CdS-6h, the (002) diffraction peak of CdS-4h is significantly enhanced, indicative of that the CdS-4h are better growth along the (002) direction.

Fig. S2 shows Uv-vis absorbance in wavelength 300 nm-800 nm of samples CdS-T-1h, CdS-T-2h, CdS-T-3h and CdS-T-4h. All curves show good visible light absorption of CdS, and the absorption edge is approximately 550 nm, corresponding to 2.25 eV in the band gap.

XRD pattern in Fig. S3 also displays the wurtzite structure for the CdS-HT-3h. Compared to XRD patterns of CdS-4h and CdS-T-3h, the (100) and (101) diffraction peaks of CdS-HT-3h is significantly enhanced, indicating that branches grew succesffully on the surface of CdS backbone.

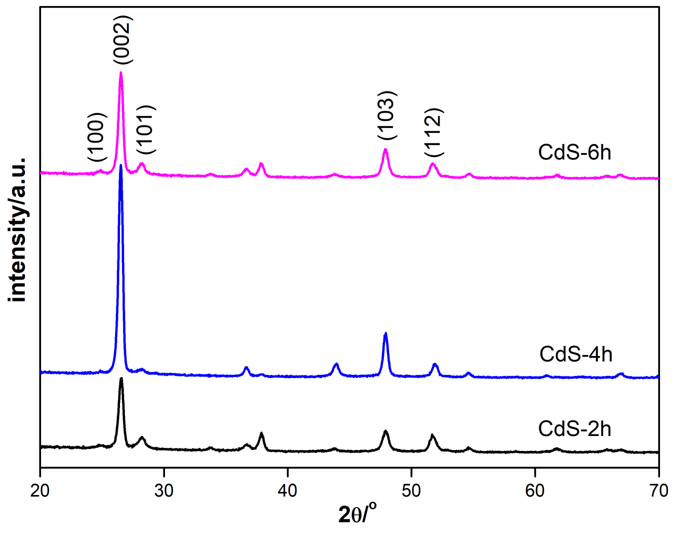

Figure S1. The XRD patterns of samples CdS-2h, CdS-4h and CdS-6h

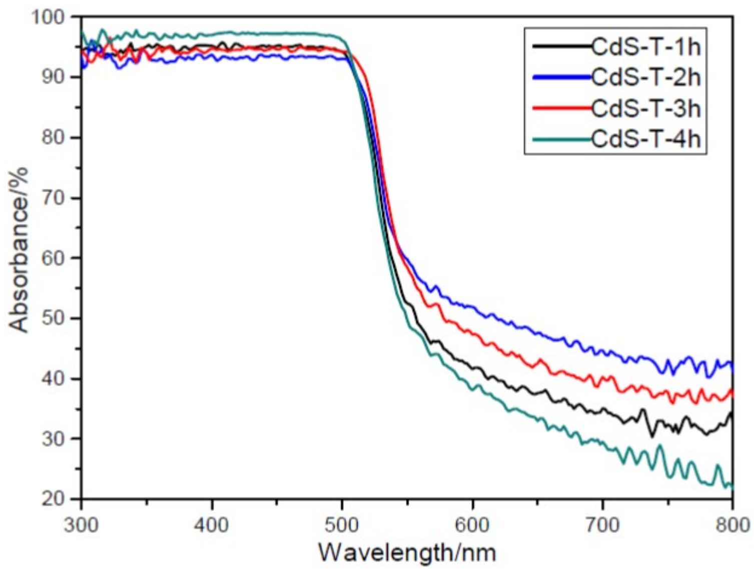

Figure S2. Uv-vis absorbance in wavelength 300 nm-800 nm of samples CdS-T-1h, CdS-T-2h, CdS-T-3h and CdS-T-4h

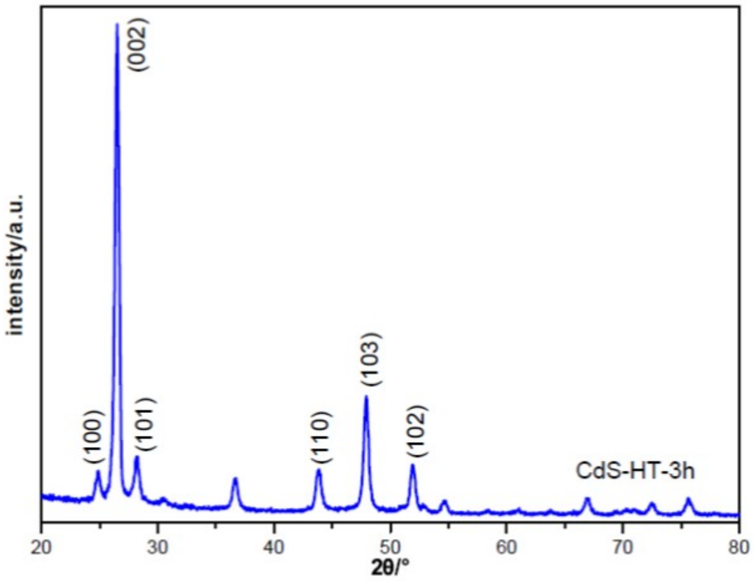

Figure S3. The XRD pattern of sample CdS-HT-3h
